# How watching sports events empowers people’s sense of wellbeing? The role of chain mediation in social interaction and emotional experience

**DOI:** 10.3389/fpsyg.2024.1471658

**Published:** 2024-12-06

**Authors:** Jiru Guo, Hong Yang, Xiaoli Zhang

**Affiliations:** ^1^School of Economics and Management, Shanghai University of Sport, Shanghai, China; ^2^School of Physical Education, Xi’an Physical Education University, Xi’an, China; ^3^School of Physical Education and Sport Science, Fujian Normal University, Fuzhou, China

**Keywords:** watching sports events, subjective wellbeing, social interaction, emotional experience, chain mediation

## Abstract

**Background:**

While engaging in sports is widely recognized for enhancing wellbeing, limited research has examined the effects of watching sports events on individuals’ subjective wellbeing. The mechanisms and pathways underlying this relationship remain unclear.

**Objectives:**

This study explores the correlation between watching sports events and the wellbeing of Chinese individuals, based on the theoretical framework of “spectator behavior → social interaction → emotional experience → happiness.” The aim is to investigate the mediating effects of social interaction and emotional experience, providing insights for promoting greater participation in sports events and supporting the healthy development of the sports industry.

**Methods:**

The study involved 885 participants from five representative provinces and cities in China. Assessment tools included the Physical Activity Rating Scale, Social Interaction Questionnaire, Emotional Experience Questionnaire, and Subjective Wellbeing Scale. Data were analyzed using Stata and the PROCESS plug-in of SPSS for comprehensive multivariate statistical analysis.

**Results:**

Watching sports events significantly and positively affects subjective wellbeing, social interaction, and emotional experience (*p* < 0.001). Three mediating pathways were identified: (1) watching sports events → social interaction → subjective wellbeing (effect size: 0.024), (2) watching sports events → emotional experience → subjective wellbeing (effect size: 0.011), and (3) watching sports events → social interaction → emotional experience → subjective wellbeing (effect size: 0.003).

**Conclusion:**

The direct impact of watching sports events on subjective wellbeing was positive. Indirect effects were facilitated by the mediating roles of social interaction and emotional experience, with the effect of social interaction being more substantial than that of emotional experience.

**Implications:**

These findings suggest that watching sports events can serve as a catalyst for enhancing wellbeing, primarily through fostering social connections and enriching emotional experiences. Practically, this indicates the potential value of encouraging viewership of sports events as a means of promoting community engagement and mental health, thus contributing to the holistic growth of the sports sector and public health initiatives.

## 1 Introduction

Enhancing the wellbeing of individuals is a universally sought-after objective (Frijters et al., 2020; Layard, 2021). Participation in sports is acknowledged as a crucial avenue to attain this goal (Silva et al., 2019; Ming, 2020; Li and Huang, 2024; Khan et al., 2023). Watching sports events, an essential component of sports involvement, can evoke communal joy (Yin et al., 2023), significantly boosting individuals’ wellbeing. Following the conclusion of the COVID-19 pandemic, sporting events globally swiftly resumed, and the audience’s excitement for watching sports events experienced a notable resurgence. In comparison to 2022, the viewership of the National Football League (NFL) in the United States saw an almost 10% increase in 2023, both on television and in live attendance (Kerai, 2023). The viewership of NBA games in 2023 continued to rise, reaching a new pinnacle with a total of 22,538,518 viewers for the 2023–24 regular season (NBA Communications, 2024). The 2023 Asian Games in Hangzhou, China, garnered the highest television ratings to date (Central Video, 2023), and the Paris 2024 Olympic Games are anticipated to generate substantial viewer interest. Given this context, exploring “how the act of watching sports events enhances individuals’ wellbeing” holds significance and relevance at the present time.

Sports participation can be categorized into direct involvement, such as engaging in physical activities and sports, and indirect involvement, which includes watching sports and consuming sports content (Yuanzhen, 2001). While many studies have focused on the benefits of wellbeing from direct sports participation (Skibinska et al., 2021; Harvey et al., 2018; Yong and Ling, 2021), the connection between watching sports events, which is an indirect form of participation, and wellbeing has not received sufficient attention from researchers (Lera-López et al., 2021). Some studies have indicated that watching sports events can elicit happiness through feelings of joy, excitement, and relaxation. However, these studies have often been confined to theoretical discussions (Yucheng and Yanzhi, 2020) or have targeted specific audiences, such as Bundesliga soccer match spectators (Götz et al., 2020; Jang et al., 2017), CBA fans (Xiaolin, 2023), and individuals with disabilities. While it has been suggested that observing sports matches can improve wellbeing (Kinoshita et al., 2024), the extent of this improvement, the mechanisms, and accessory pathway involved remain unclear. Moreover, China, being the most populous nation globally, consistently boasts the highest number of sports viewers. For instance, during the 2022 Winter Olympics in Beijing, the Chinese audience reached a peak, with nearly 600 million viewers in the initial week (Statista, 2020). Nonetheless, there exists a notable research void regarding how the average Chinese viewer enhances their wellbeing through watching sports events.

According to Randall Collins’ Interaction Ritual Chain Theory (Hallett, 2003), watching sports events establishes a dynamic setting where spectators partake in an exchange of energy. Viewers interact socially as a result of common interests in individuals, events, and subjects. Furthermore, they undergo varying emotions shaped by the unfolding developments of the events. These social exchanges evoke emotional reactions, culminating in a state of “collective euphoria” and a profound feeling of wellbeing.

Building upon previous studies, this research undertakes a comprehensive survey in China with dual objectives: firstly, to validate the direct boosting effect of watching sports events on the happiness of individuals in China, and secondly, to investigate the mediating and transmitting mechanisms involving social interaction and emotional experiences within this correlation. The primary aim of this study is to enhance the theoretical frameworks and uncover the fundamental influence mechanisms. Furthermore, it aspires to foster worldwide interest in engaging in sports events, thereby fostering the wholesome advancement of sports on a global level.

### 1.1 Chain mediation

Chain mediation refers to a specific type of mediation model where two or more mediating variables are linked sequentially, forming a chain-like process through which the independent variable affects the dependent variable. In a chain mediation model, the effect of the independent variable is transmitted through multiple mediators in a specified order, highlighting the stepwise progression of influence (Zhao et al., 2022). For instance, in this study, the impact of watching sports events on subjective wellbeing is first mediated by social interaction, which in turn influences emotional experience, ultimately affecting wellbeing.

### 1.2 The relationship between watching sports events and wellbeing

The happiness associated with watching sports events stems from the aspects of these events that evoke positive emotions like happiness, excitement, joy, pride, and satisfaction (Yucheng and Yanzhi, 2020). Experiencing these emotions triggers subjective happiness (Lee et al., 2014). Japanese researchers have validated this through the analysis of open big data, online surveys, and MRI neuroimaging experiments, demonstrating simultaneously that watching sports events improves wellbeing (Kinoshita et al., 2024). Particularly, wellbeing reaches its peak when viewers witness their favorite teams emerge victorious. For example, Stieger et al. (2015) observed that fans of the 2014 World Cup reported increased subjective wellbeing following their national team’s success. Macrocosmically, hosting sports events also enhances the wellbeing of the local populace. Hosting sports events, such as the Paris 2024 Olympics, is expected to have mixed impacts on local wellbeing. While there are notable infrastructural improvements and increased economic activity in some areas, it also brings challenges for marginalized communities, with increased evictions and social tensions (Paris, 2024).

*RH1*: Watching sports events can directly contributes to enhancing individuals’ wellbeing.

### 1.3 The mediating role of social interactions between watching sports events and wellbeing

Watching sports events can also encourage social engagement and promote positive interpersonal relationships. Sports events usually attract large audiences, creating a conducive environment for socializing, sharing experiences, and fostering communication. Whether among family, friends, or within broader communities, paying attention to sports events together and participating in them can facilitate mutual understanding, strengthen bonds, and enhance social aptitude, and thus access to more social resources (Keyes et al., 2023). Additionally, watching sports events nurtures a sense of community belonging and collective cohesion. In these settings, individuals come together in support of common objectives or national pride, reinforcing community or national solidarity and forging wider social connections. This, in turn, cultivates trust and wellbeing among individuals (Lin and Liu, 2020). Meanwhile, social interactions, which is also crucial for wellbeing, can provide a buffer and social support system to help people cope with the stress and challenges in life. Sharing emotions and concerns with peers, family, or the community helps alleviate psychological burdens, reduce stress, and cultivate feelings of acceptance and support, ultimately enhancing feelings of security and belonging (Acoba, 2024).

*RH2*: Social interactions play an intermediary role in enhancing individuals’ wellbeing during watching sports events.

### 1.4 The mediating role of emotional experience between watching sports events and wellbeing

Watching sports events can evoke a wide range of emotional responses, contributing to overall wellbeing. Research shows that viewing sports can trigger positive changes in both subjective wellbeing and brain activity. A study using neuroimaging methods found that participants experienced heightened brain activation in reward-related areas when watching sports, indicating that it can induce feelings of happiness and pleasure. These effects are further intensified by watching popular sports such as baseball or soccer, which also lead to positive structural changes in the brain over time (Kinoshita et al., 2024). The intricacy of movements, the intensity of competition, the variety of forms, and the unpredictability of results lead to strong emotional reactions in both participants and onlookers (Fengzhen et al., 2007). Spectators commonly feel a mix of emotions including excitement, frustration, exhilaration, happiness, anger, admiration, and surprise. The feeling of joy that arises when a favored team or player triumphs can bring about a surge of happiness that endures well beyond the event’s end (Jang et al., 2018; Jang et al., 2017; Stieger et al., 2015). Positive and beneficial emotional encounters can directly contribute to individuals’ happiness. When viewers experience positive emotions like happiness, contentment, and pride during watching sports events, these feelings heighten their enjoyment and contentment, thereby enriching their overall happiness.

*RH3*: Emotional experiences play a mediating role in enhancing people’s wellbeing during watching sports events.

### 1.5 Chain mediation of social interactions and emotional experiences between watching sports events and wellbeing

The theory of interactive ritual chain emphasizes the central role of social interaction in the formation of emotional experiences (Collins, 2004). This theory suggests that individuals establish shared emotional experiences through participating in specific rituals together in social interactions. Ultimately, frequent participation in interactive rituals can accumulate lasting emotional connections, which in turn enhance an individual’s sense of happiness and life satisfaction. When people watch sports events, the audience cheers, encourages, or loses together. Each interactive ceremony may strengthen the emotional resonance of participants, making them feel deeper emotional investment when facing joy or setbacks. Therefore, the theory of interactive ritual chain provides an important perspective for understanding how social interaction affects emotional experiences. Hallett (2003) also proposed that social interactions act as triggers for emotional responses. This suggests that social interactions play a role in enriching individuals’ emotional experiences. In the study by Ye and Linsheng (2020), they analyzed the impact of sports engagement on subjective wellbeing, and described that the influence of watching sports events on wellbeing can be depicted as a pyramid. The solid base of the pyramid is the behavior of the audience watching the sports competition in a healthy way, the advance level is the good social interaction and communication, and the spire layer is the acquisition of the emotion (beauty, etc.) of the game.

*RH4*: A chain-mediated effect exists between social interactions and emotional experiences in enhancing individuals’ wellbeing during watching sports events.

**Figure 1:**
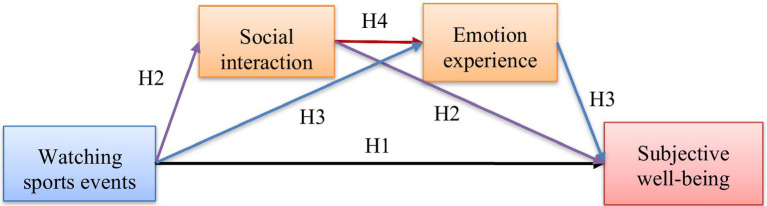
Theoretical model.

As a result, this research has formulated a chain model that integrates social interactions and emotional experiences as mediating factors. The purpose of this model is to elucidate how observing sports events contributes to individuals’ wellbeing, as depicted in Figure 1.

## 2 Methods

### 2.1 Participants

The information utilized in this manuscript is derived from a nationwide survey carried out by the China National Social Science Foundation Program between 2021 and 2022. The survey primarily aimed at urban dwellers in China, ranging from 18 to 80 years old. The questionnaire development process was segmented into three phases: initial design, pre-survey, adjust and finalize. The survey encompassed five urban areas across China, including Shanghai in the east, Zhengzhou, Changchun in central China, and Xi’an and Liupanshui in western China. A total of 1,600 questionnaires were disseminated, yielding 1,343 valid responses. This study involves watching sports events, among which 885 people have watching experience, which is the research object of this paper. Prior to questionnaire completion, all participants provided signed and informed consent forms, and all procedures were in accordance with the ethical guidelines of the Research Committee of Xi’an Physical Education University.

### 2.2 Materials

#### 2.2.1 Basic personal information

The basic personal information section of the questionnaire included questions on gender, age, education level, health status, income, etc.

#### 2.2.2 Independent variable: watching sports events

Watching sports events is an indirect way to participate in sports. With reference to the “Sports Activity Rating Scale” revised by Deqing (1994), the frequency and duration of watching sports events are mainly used to measure (because the intensity of watching sports events is difficult to measure). Watching sports events is segmented into “on-site viewing” and “media viewing.” Consequently, the measurement questions include: (1) How often do you usually watch sports events through media channels such as TV, Internet, cell phone, computer, or tablet computer? Answer choices: A. Once a month or less B. Twice to three times a month C. Once to twice a week D. Three to five times a week E. About once a day, each assigned a value from 1 to 5 points. (2) How often do you usually watch sports events in person? Answer choices are the same as above, with each choice assigned a value from 1 to 5 points. (3) How long do you typically watch a sports game? Answer choices: A. 10 min or less B. 11–20 min C. 21–30 min D. 31–59 min E. 60 min or more, each assigned a value from 0 to 4 points. The study defines the measurement formula as the sum of the frequency of live viewing and viewing frequency through media multiplied by viewing duration, treating it as a continuous variable.


$$f\left(x\right)=\left(\begin{array}{l} \mathrm{the frequency of live viewing}+\\ \mathrm{viewing freuency through media} \end{array}\right)*\mathrm{viewing duration}$$

#### 2.2.3 Mediating variables: social interaction, emotional experience

All the questions in the social interaction and emotional experience questionnaire used Likert scale, and the answer options were divided into: “completely disagree, disagree, uncertain, agree, completely agree,” with 1–5 points, respectively. The Social Interaction Scale drew from the metrics outlined by Wann and James (2018) and Stieger et al. (2015), using four statements: (1) I aim to connect with more individuals through sports observation. (2) I seek to enhance my social engagements through sports viewing. (3) I derive pleasure from spending time with sports enthusiasts. (4) I prefer watching sports events with friends for the enhanced ambiance it creates. Scores for each statement were aggregated and treated as a continuous variable. Concerning emotional experience, the study referenced Scherer (2005) criteria, combining the characteristics of sports viewing. The emotional experience section contains three measurement questions, that investigated the individual’s emotional mobilization, excitement and aesthetic feeling in the process of watching the games respectively, which are: (1) Watching a sports event stirs my emotions. (2) Sports viewing fills me with excitement. (3) Watching sports events provides me with a sense of beauty. Scores for these statements were likewise totaled and handled as continuous variables.

#### 2.2.4 Dependent variable: subjective wellbeing

The investigation utilizes the Subjective Wellbeing Scale (SWB) developed by Diener (1984) and the Abbreviated Version of the Subjective Wellbeing Scale for Urban Dwellers in China by Zhanjun (2003) to assess subjective wellbeing across four facets: overall assessment of wellbeing, life contentment, positive emotions, and negative emotions. The assessment queries are as follows: (1) Do you feel happy with your life now? A. Extremely unhappy B. Unhappy C. Average D. Happy E. Extremely happy. (2) How satisfied are you with your life? A. Extremely dissatisfied B. Dissatisfied C. Average D. Satisfied E. Extremely satisfied. (3) Do you perceive life as engaging? A. Not at all B. Very minimally C. Engaging D. Quite engaging E. Extremely engaging. (4) Are you experiencing negative emotions (e.g., sadness, hopelessness, worry, gloom)? A. Always B. Frequently C. Occasionally D. Rarely E. Never. Each question’s five choices were assigned a value from 1 to 5. The totals for each query were aggregated to form a continuous variable for subjective wellbeing.

#### 2.2.5 Control variables

Previous research (Xiaoli et al., 2019; Qian et al., 2019; Xiaoli et al., 2022) has indicated that socio-demographic and socio-economic factors play a role in shaping individuals’ subjective wellbeing. Therefore, these factors were chosen as control variables for the regression model analysis. They were defined and categorized as follows: Gender: male = 0, female = 1. Age: young (18–39 years) = 1, middle-aged (40–60 years) = 2, old (60 years and above) = 3. Education level: high school and below = 0, specialized/undergraduate = 1, graduate and above = 2. Health status: unhealthy = 0, general = 1, healthy = 2. Monthly income: low-income (0–3,000 yuan) = 0, middle-income (3,001–8,000 yuan) = 1, high-income (above 8,000 yuan) = 2.

### 2.3 Reliability and validity test of the scale

During the design and development phase of the scale, all questionnaire items were referred to established scales sourced from both domestic and international origins, with minor adjustments tailored to the specific context of watching sports events in China. After several discussions by the members of the research group, and interviews with experts and survey objects, the scale was finally designed. The reliability of the scale was evaluated using SPSS 26.0, with Cronbach’s *α* coefficients for the four variables surpassing 0.7, indicating four scales have good consistency and high reliability. Furthermore, Stata 16.0 was utilized to examine the content validity of the scale. The χ^2^/df values ranged from 1 to 8, while metrics such as GFI, AGFI, CNFI, NNFI/TLI, and IFI all exceeded 0.9, and the RMSEA was below 0.001. These findings suggest that the model’s fit indices were satisfactory, affirming the scale’s robust content validity. Detailed outcomes are omitted for conciseness.

## 3 Results

### 3.1 Descriptive statistics of main variables

The descriptive statistical results for the independent, mediating, dependent, and control variables are detailed in Table 1.

**Table 1:** Descriptive statistical results of the main variables.

Variable name		M ± SD	Control variables		Percentage occupied
Independent variables	Watching sports events	12.147 ± 6.029	Education	High school and below	20.45%
Mediating variables	Social interaction	9.769 ± 2.771		Specialized/Undergraduate	62.39%
	Emotion experience	5.877 ± 2.415		Graduate students and above	16.16%
Dependent variables	Subjective wellbeing	14.491 ± 1.243	Health	Healthy	87.80%
Control variables		Percentage held		General	10.73%
Gender	Men	56.84%		Unhealthy	1.47%
	Female	42.16%	Income	Low-income	40.05%
Age	Youth	42.66%			
	Middle-age	26.13%		Middle-income	42.50%
	Old	31.32%		High-income	16.45%

### 3.2 Common method bias and collinearity diagnostics

The Harman single-factor test was utilized to examine potential common method bias. Initially, an exploratory factor analysis was conducted on the independent, mediating, and dependent variables, revealing four common factors with initial eigenvalues that exceeding 1. These factors jointly accounted for 69.83% of the variance, with the primary factor (watching sports events) explaining 23.42% (below the 40% threshold). This outcome suggests the absence of significant common method bias within the survey data. Subsequently, tolerance and variance inflation factor (VIF) assessments were carried out on the independent, mediating, and control variables. Tolerance values fell within the range of 0.36–0.45, all surpassing the 0.1 threshold. VIF values ranged from 3.02 to 3.71, all below 10, indicating the absence of multicollinearity concerns among these variables.

### 3.3 Direct influence of watching sports events on enhancing subjective wellbeing

In this study, watching sports events was examined as the independent variable, while subjective wellbeing served as the dependent variable. The regression model incorporated control variables such as gender, age, education, health, and income. To assess the direct impact of watching sports events on subjective wellbeing, an Ordinary Least Squares (OLS) regression analysis was conducted using SPSS 26.0. The findings, detailed in Table 2, demonstrate a standardized regression coefficient of 0.160 for watching sports events on subjective wellbeing, significant at *p* < 0.001. This suggests that watching sports events positively influences individuals’ subjective wellbeing, even when control other factors. As a result, RH1 is supported.

**Table 2:** Main effect test of watching sports events on enhancing people’s subjective wellbeing.

Predictor variables	Model 1 subjective wellbeing
Independent variables	
Watching sports events	0.160***
Control variables	
Gender	0.067
Age	0.056
Education	0.035
Health	0.144***
Income	0.040
*F*	8.091
*R* ^2^	0.052

*p < 0.05, **p < 0.01, ***p < 0.001; F means F-statistic, which assesses the overall significance of the regression model; R^2^ means Coefficient of Determination, showing the proportion of total variance in the dependent variable that is explained by the model. The same meanings are applied in the following tables. The control variables used in the subsequent Tables 3, 5 are the same as those in this table. In order to better present the results, the control variables in the subsequent tables have been simplified.

### 3.4 Indirect effects of watching sports events on subjective wellbeing

To delve deeper into the mediating roles of “social interaction” and “emotional experience” in the correlation between watching sports events and subjective wellbeing, a stepwise regression analysis was first executed. Subsequently, bootstrap method was further used to analyze the mediating effect.

#### 3.4.1 Analysis of the mediating effect of social interaction

The outcomes of the regression analysis concerning the mediating impact of social interaction are outlined in Table 3. Initially, with social interaction as the dependent variable, watching sports events and control variables were placed in the regression model 2. The analysis unveiled a noteworthy positive influence of watching sports events on social interaction. Subsequently, in regression model 3, subjective wellbeing was designated as the dependent variable. Watching sports events, social interaction, and control variables were all simultaneously integrated into the model. The results highlighted a significant positive impact of social interaction on subjective wellbeing. Furthermore, the standardized regression coefficient for watching sports events remained positive. Nevertheless, compared with the regression analysis of watching sports events in Model 1 from Table 2, the standardized regression coefficient decreases from 0.160 to 0.127. This decline indicates a partial mediating effect of social interaction in the relationship between watching sports events and subjective wellbeing.

**Table 3:** Testing the mediation effect of social communication.

Predictor variables	Model 2 social interaction	Model 3 subjective wellbeing
Independent variables		
Watching sports event	0.371***	0.127**
Social interaction		0.088*
Control variables	Already controlled	
*F*	28.374***	7.851***
*R* ^2^	0.162	0.059

*p < 0.05, **p < 0.01, ***p < 0.001.

To better evaluate the magnitude of the mediating effect of social interaction, model 2 from the PROCESS plug-in in SPSS was utilized, and the test was carried out by the bootstrap method. The findings are presented in Table 4. This shows that the direct effect of watching sports events on subjective wellbeing is 0.093, with a 95% confidence interval excluding 0. So the direct effect is significant, which represents 79.5% of the overall effect size. Additionally, the mediating effect of social interaction is 0.024, with its 95% confidence interval also excluding 0, indicating that the indirect effect is significant and accounts for 20.5% of the total effect. Consequently, watching sports events not only directly enhances subjective wellbeing but also indirectly improves it through social interaction which is an intermediate path. Thus, both research hypotheses H1 and H2 are validated.

**Table 4:** Analysis of the contribution rate of intermediary effects in social communication.

Impact pathways	Effect value	Effect proportion	Boot SE	95%CI		Hypothesis testing
				Lower bound	Upper bound	
Total effect	0.117***	100.0	0.025	0.068	0.165	
Direct effect (watching sports events → subject wellbeing)	0.093**	79.5	0.027	0.040	0.145	H1 is supported
Indirect effect (watching sports events → social interaction → subject wellbeing)	0.024**	20.5	0.010	0.003	0.044	H2 is supported

Boot SE means Boot Standard Error, 95%CI means 95% Confidence Interval. The same meanings are applied in the following tables. *p < 0.05, **p < 0.01, ***p < 0.001.

#### 3.4.2 Analysis of the mediating effects of emotional experience

Following the methodology of the social interaction research, the first step was to conduct a mediating regression analysis of emotional experiences, the outcomes of which are outlined in Table 5. Subsequently, an examination of this mediating effect was undertaken, with findings presented in Table 6. Model 4 in Table 5 reveals a noteworthy enhancement in emotional experiences induced by spectating sports events, demonstrated by a standardized regression coefficient of 0.154 (*p* < 0.001). Table 5 Model 5 depicts a significant impact of enhanced emotional experiences on subjective wellbeing, affirmed through a standardized regression coefficient of 0.093 (*p* < 0.005). Yet, when juxtaposed with the standardized regression coefficient rendered from the watching sports events study within Model 2 in Table 3, there was a decrease from 0.160 to 0.146. This reduction suggests emotional experience partially intermediates the relationship between watching sports events and subjective wellbeing.

**Table 5:** The mediating effect test of emotional experience.

Predictor variables	Model 4 emotional experience	Model 5 subjective wellbeing
Independent variables		
Watching sports event	0.154***	0.146***
Emotional experience		0.093**
Control variables	Already controlled	
*F*	3.010***	8.111
*R* ^2^	0.027	0.061

*p < 0.05, **p < 0.01, ***p < 0.001.

**Table 6:** Analysis of the mediation effect contribution rate of emotional experience.

Impact pathways	Effect value	Effect proportion	Boot SE	95%CI		Hypothesis testing
				Lower bound	Upper bound	
Total path	0.117***	100.0	0.025	0.068	0.165	
Direct effect (watching sports event → subject wellbeing)	0.106**	90.6	0.025	0.057	0.155	H1 is supported
Indirect effect (watching sports event → emotional experience → subject wellbeing)	0.011**	9.4	0.005	0.003	0.020	H3 is supported

*p < 0.05, **p < 0.01, ***p < 0.001.

In order to more precisely determine the magnitude of the mediating effect of emotional experience, the bootstrap method was implemented using Model 4 of the PROCESS plug-in in SPSS with the outcomes indicated in Table 6. The direct impact of watching sports events on subjective wellbeing is 0.106, the 95% confidence interval of which excludes 0, demonstrating that the direct effect is statistically significant and accounts for 90.6% of the total effect size. The value of the mediating effect of emotional experience is 0.011, and just like the direct effect, its 95% confidence interval also excludes 0, denoting a significant indirect effect that represents 9.4% of the total effect. As a result, it can be inferred that watching sports events can directly elevate people’s subjective wellbeing and indirectly affect it through the mediating route of emotional experience. Hence, both H1 and H3 research hypotheses are solidly corroborated.

#### 3.4.3 Analysis of chain-mediated effects of social interaction and emotional experience

In utilizing Model 6 within the PROCESS plug-in of SPSS, the bootstrap method was executed to scrutinize the chain mediation effects of both social interaction and emotional experience. The related findings are displayed in Table 7. The indirect influence of these dual factors is marked at 0.030, with its accompanying confidence interval excludes 0, signifying a noteworthy aggregate indirect effect, contributing to 25.6% of the total effect. The indirect roles of the following sequences, “watching sports events → social interaction → wellbeing” and “watching sports events → emotional experience → wellbeing” endure as significant, with their respective effect proportions pegged at 17.9 and 5.1%. Additionally, the chain mediation sequence of “viewing sports events → social interaction → emotional experience → wellbeing” which was mainly focused on registers an influence value of 0.003 and a confidence interval that does not intersect 0, hence affirming its significant chain mediation effect that represents 2.6% of the total indirect effect. As a result, a chain mediation effect involving social interaction and emotional experience significantly escalates people’s subjective wellbeing through watching sports events. The mediation effect exerted by social interaction overshadows that of emotional experience, thereby substantiating RH4.

**Table 7:** Chain mediation testing of social communication and emotional experience.

Impact pathways	Effect size	Effect proportion	Boot SE	95%CI		Hypothesis testing
				Lower bound	Upper bound	
Total path	0.117***	100.0	0.025	0.068	0.165	
Direct effect	0.087**	73.4	0.027	0.034	0.139	
Indirect effect	0.030**	25.6	0.011	0.010	0.051	
Watching sports event → social interaction → subject wellbeing	0.021***	17.9	0.011	0.000	0.041	H2 is supported
Watching sports event → emotion experience → subject wellbeing	0.006**	5.1	0.003	0.000	0.014	H3 is supported
Watching sports event → social interaction →emotion experience → subject wellbeing	0.003**	2.6	0.002	0.001	0.008	H4 is supported

*p < 0.05, **p < 0.01, ***p < 0.001.

## 4 Discussion

### 4.1 The relationship between watching sports events and subjective wellbeing

This study’s findings illustrate that viewing sports events have a direct and positive impact on individual subjective wellbeing, manifested by a standardized regression coefficient of 0.160. This impact is highly substantial (*p* < 0.001), thereby addressing the initial research hypothesis: watching sports events can directly uplift people’s subjective wellbeing, and the enhancement effect is relatively significant. This auspicious outcome reinforces the idea that not just active sports participation (like engaging in physical activities) can promote wellbeing but indirect (or what is referred to as passive) forms can yield similar results. This conclusion aligns with the studies carried out by Spanish researchers suggesting that various passive sports participation forms, such as watching sports games, listen to sports programs, and scanning sports news, display strong correlation with wellbeing (Lera-López et al., 2021). Irrespective of whether it’s via live attendance or media broadcast, sports viewership inherently yields stimulation, excitement, and delight. This endeavor allows a respite from daily life burdens and work stress, enabling individuals to unwind physically and psychologically, reduce stress and irritation, and immerse themselves—visually, audibly, etc.—in a dynamic experience. From the release of the body to the activation of the brain’s excitatory system, people can experience the sense of fluency, pleasure and orgasm brought by sports, the integration of the sense of beauty and excitement, and finally reached the immersive experience of extreme happiness. This shows that watching sports events itself is the main event and source of wellbeing, and it also has important implications in policy formulation. When the national fitness craze is in full swing, how to further improve the required conditions for watching sports events, enhance the enthusiasm for watching sports events, use sports events to enable residents to feel the sense of wellbeing, and create a harmonious, healthy, positive and relaxed social atmosphere is also an important proposition that cannot be ignored.

### 4.2 Mediating role of social interaction

Beyond relaxation and entertainment, increasing social interaction is a significant motivation for watching sports events. “Shared interests and sympathies” naturally foster social interactions in this enthusiastic atmosphere. Occurrences of such fellowship can be traced to watching matches with family or friends, cheering alongside fans in-stadium, or being a part of social clusters such as fan clubs that indulge in consistent interactions. Regular contact within these social assemblies solidifies connections between members unified by common subjects and pastimes. Studies reveal that involvement in sports, primarily during recreative periods, provides venues for social interaction and encourages individuals in honing their communicative and collaborative-abilities, thereby contributing to more fulfilling lives (Bloodworth et al., 2012). Interacting with family, friends, neighbors, fellow fans, and sports teams allows individuals to fortify existing social ties, engineer new ones, and uncover heightened feelings of social belonging and identity (De Haan et al., 2014; Stander et al., 2016; Zhou and Kaplanidou, 2018). These interactions stimulate emotional reciprocation, knowledge exchange, and emotional unity. Social interactions also serve as solid predictors of wellbeing. Individuals exhibiting sociability and extroversion rationally find more happiness than those less sociable. Those who dedicate relatively larger durations with others experience more happiness than those who lean toward solitude, and ones with an extensive friendship circle logically exhibit more happiness than those with only a few friends. Therefore, the camaraderie, backing, associations and interactions originating from sports viewership are noteworthy origins of happiness.

### 4.3 The mediating role of emotional experience

As posited by Diener (1984), emotional states can be bifurcated into positive emotions (such as exhilaration, delight, and relaxation) and negative emotions (including stress, fury, and melancholy). Studies suggest that positive emotional experiences are pivotal in attaining wellbeing. Conversely, venting negative emotions could be equally efficacious (Shiota et al., 2017). Watching sports events serves as a platform satisfying both the amplification of positive emotions and the purge of negative emotions. This emotionally charged experience is key to sourcing a feeling of wellbeing. Guided by the permutations of the sports teams’ various performance, viewers might engage in flag-waving, cheering, expressing dismay, or manifesting nervousness and irritability. These intense emotional experiences pervade the whole tenure of watching sports broadcasts. This allows individuals a means to dispense with negative emotions, alleviate life’s tensions, and indulge in gratification at both physical and mental levels. This procedure incrementally morphs into positive emotions, encompassing happiness and satisfaction, ultimately fostering an enriched sense of wellbeing.

### 4.4 Chain mediating role of social interaction and emotional experience

The range of human needs is structured in a hierarchy, extending from basic to advanced levels. Beyond primal necessities such as food, beverage, and sleep, recreational and entertainment activities constitute the bedrock of life’s needs. Sports event viewership acts as a crucial source of such leisure and amusement. However, its sustained allure stems from satisfying superior demands, among them being social interaction and emotional experience. Firstly, the act of consuming sports cultivates a milieu for social interactions and facilitates the accrual of social resources. Social interplays considerably shape people’s emotional experiences, as sports viewership is, by its very nature, a social act. Persons with akin interests have the opportunity to co-experience their emotional states. While viewing a match in the company of friends, family, or colleagues, they can exchange perspectives and sentiments and collectively experience the match’s triumphs, disappointments, and suspenseful moments. This communal emotional experience deepens their bond and amplifies their resonance, contributing to greater pleasure and satisfaction derived from the game. Additionally, spectators can achieve a sense of identity via fan associations or the members of a jointly supported team while watching sports events. This feeling of social identity can elevate self-assurance and impart a sense of belonging, thereby enriching the emotional experience. Healthy social interactions and positive emotional experiences produced within sports viewership spaces significantly elevate the sense of wellbeing.

While both social interplay and emotional experience serve as key mediating factors, social interaction wields a stronger mediating force compared to emotional experience. This can be deciphered through the notions of “transient effects” and “long-lasting effects.” The enhancement of social support and relationship network brought by social interaction enhances the breadth of social support, delivering a more comprehensive influence on wellbeing. This is a long-term, sustained positive effect, and the improvement of subjective wellbeing has a lasting effect. Conversely, emotional experience is typically ephemeral, yielding quick-lived gratification. Though capable of presenting intense emotional gratification, this profound experience typically necessitates the support of other factors, such as enduring social interplay, to cultivate a lasting sense of wellbeing. Consequently, its contribution to subjective wellbeing tends to be more ephemeral. This implies that for durable happiness derived from sports viewership, lasting social interplays yield greater dividends compared to transient emotional pleasure. Profound interpersonal bonds and spiritual exchanges form the core sources of happiness.

### 4.5 How positive viewing experiences promote participation in physical activity

Positive viewing experiences can stimulate people’s interest and motivation to engage in physical activity (Fleshman and Kaplanidou, 2023). The joy and sense of happiness brought by watching sports events may encourage spectators to try participating in physical activities, especially when they experience the athletes’ determination and enjoyment in the competition. This emotional experience is not limited to the viewing process but can inspire people to extend these emotions into their daily lives, thereby becoming more actively engaged in physical exercise. Particularly when spectators feel emotionally devoted and fulfilled, they may develop a stronger intention to engage in physical activity, which may manifest in both short-term and long-term participation after the event. In summary, positive viewing experiences not only bring emotional joy but also serve as a source of motivation, driving individuals to adopt a healthier lifestyle (Inoue et al., 2015).

### 4.6 Limitations and future studies

Although this paper explores the relationship between watching sports events and happiness and the corresponding mechanism, there are the following areas that need to be improved. (1) The breadth of the study’s sample could be extended. The current sample includes 885 respondents from the east, center, and west regions of China. However, encompassing a larger sample could boost the general applicability of the conclusions. (2) The measurement indicators of watching sports events need to be further enriched. The measurement indicators used in this study only include the frequency and duration of watching sports events, but lack measurement of viewing intensity. Future research should explore better measurement questions for the intensity of watching sports events, such as “emotional engagement in watching games.” In addition, these measurement indicators focus more on the external behavior of watching sports events, while the motivation, type and way of watching sports events need to be further studied and refined to form a mature measurement scale for watching sports events. (3) Compare the effects of varying forms of watching sports events on wellbeing. This manuscript does not differentiate between the effects that online and offline sports viewership have on happiness. Subsequent examinations could utilize models like coefficient clustering to analyze this distinction. (4) Delve into more intricate influence mechanisms and relational paths. Given that the genesis of happiness encompasses many variables, this study concentrates exclusively on the mediation roles of social interaction and emotional experience. Subsequent research should incorporate more variables to probe these multifaceted mechanisms. (5) While this study focuses on the impact of sports viewership on subjective wellbeing, other contextual factors, such as workplace environment and social support, are also crucial contributors to wellbeing. Research suggests that a positive work environment and strong social support networks can enhance emotional wellbeing, reduce stress, and foster greater life satisfaction. These unexamined variables are important for understanding the broader influences on subjective wellbeing. Future research should adopt a multi-context, multi-variable approach to explore how various contextual factors interact and contribute to wellbeing, offering a more comprehensive view of the mechanisms underlying happiness” (Bergefurt et al., 2023; O’Hare et al., 2024).

### 4.7 Suggestion and strategies

Firstly, escalate both the malleable and rigid prerequisites for sports viewing to magnify the residents’ zeal for spectating events. (1) For the community, increase community sports watching activities and expand publicity. Harness community bulletin boards, digital media platforms, and emails for disseminating information about forthcoming sports contests. Initiate community-centered sports exercises like football, basketball, or volleyball championships to facilitate residents’ direct participation and expand their comprehension of sports contests. For people with limited sports interest or those who encounter hurdles to participation, impart applicable education and training, such as sport-related knowledge sessions or commentator translation services. (2) For event organizers, further refine the sports viewing environment and facilities. Construct or modernize sports arenas and infrastructures to create conditions for residents to secure top-notch offline spectating experiences. At the same time, ensure that the sports events are equipped with large screen TV, projection equipment and superior sound systems to ensure the quality of real-time transmission of the game. Projector equipment Ensure an obstruction-free viewing ambiance by offering ample space, comfortable seating, and convenient access, thereby catering to all residents to watch sports events, irrespective of their physical prowess or capabilities. (3) For event planners, start by intensifying sports event supervision to secure superior quality competitions to provide residents with a high level of on-site competition with both appreciation, competition and fluency. To engage more residents to spectate in person, offer discounted or free passes to students, seniors, disabled individuals, and other special demographics, consequently augmenting the spectator turnout. Also, advance online platforms by creating or collaborating with existing ones (e.g., sports livestream websites or apps) enabling residents to stream sports contests from home. The platforms should strengthen its own construction, deliver top-tier live streaming, reliable network connectivity, and intuitive navigation to enrich the viewing experience.

Secondly, the formation of social institutions such as fan groups for sports events can enrich the social interaction of spectators. (1) Support the establishment of social collectives like fan clubs for sports events. Urge sports aficionados to found or become part of local fan clubs or associations. These clubs can arrange activities like spectating games, strategizing discussions, and taking part in fan contests, providing a platform for conversation and exchange. (2) Intensify interactions and communications among club members by arranging parties and events targeted at fans. Schedule regular gatherings for fans, providing a consistent platform for communication. These congregations can include focused discussions, live match screenings, and other participatory activities to boost involvement and a sense of community. Propose manifold fan activities, such as talent competitions, riddles, predicting sports outcomes, and sharing game experiences. These activities stationed before, during, or post-matches amplify entertainment, incite participation and encourage interplay. Inspire fans to partake in communal activities like charitable collections and environmental preservation. This engagement helps establish civic responsibility and assists fans in better assimilating into their communities. (3) Urge fans to institute social media networks or web forums for regular communication and idea exchange. Advance cultural swaps among fans hailing from diverse locales to share regional customs and traditions, fostering interregional camaraderie and cooperation.

Thirdly, improve the quality of sports events and enhance the emotional experience of residents. (1) Fortify the organization and management of events to make sure they are run in a well-organized, systematic manner. This includes prevent organization, event progression, and post-event examination. Efficient management is key for the seamless operation of sporting competitions, preventing disorder and mishaps. (2) Render an ideal viewer environment and provide user-friendly transportation facilities. Assure comfortable accommodation, clear visual and audio impressions, and a lively ambiance, which encapsulates well-maintained facilities, a selection of food and drink options, and zones for entertainment. Fabricating an enjoyable spectating atmosphere allows audiences to fully savor the game. Supplementally, offering accessible transport choices such as public conveyance, ample parking space, and clean sanitation facilities promotes smooth ingress and egress, boosting the entire viewer experience. (3) Enhance the competitiveness and grandeur of sporting events to entice more viewers and provide a spirited emotional engagement. This can be realized by uplifting the competitive intensity and incorporating elements of mystery and drama. (4) Propose assorted amusement activities during the event to stimulate audience interaction and engagement. These could involve musical and dance acts, exciting draws, and more, increasing spectator gratification and entertainment. Additionally, enable interfaces for lively crowd participation, such as cheering, sign-raising, and clapping, to nurture a sense of unity and an upbeat emotional response.

## 5 Conclusion

The research deduces the following: (1) Watching sports events can directly escalate subjective wellbeing. (2) Sports event spectating broadens the scope of social engagements and magnifies positive emotional occurrences effectively. (3) Serving as individual mediators, and as a link, both social interactions and emotional experiences amplify wellbeing through the viewership of sports events. Importantly, the mediating impact of social interaction surpasses that of emotional experiences.

## Data Availability

The raw data supporting the conclusions of this article will be made available by the authors, without undue reservation.
